# Vacant Accommodation in London Workhouses

**Published:** 1914-03-28

**Authors:** 


### VACANT ACCOMMODATION IN LONDON WORKHOUSES.

A very important step towards the utilisation of the vacant accommodation in London workhouses has been taken by the Metropolitan Asylums Board during the last few weeks. The Board finds itself in need of one or more institutions with accommodation for 700 to 1,000 inmates owing to the increasing demands made upon it in connection with its old work and with the new work entrusted to it. It is, therefore, negotiating with the City of Westminster Union to acquire the workhouse at Edmonton and with the Greenwich Guardians for the Grove Park Workhouse. The Board points out that, according to the latest returns, there are over 9,000 vacant beds in the Metropolitan workhouses, that the City of Westminster has 3,324 workhouse beds, whilst the number of inmates on January 31, 1914, was only 2,319, and suggests that the Westminster and Greenwich Guardians might find accommodation for the inmates displaced from the Edmonton and Grove Park institutions in other of their own workhouses, or by boarding out their inmates with other Unions. The City of Westminster Guardians, after consultation, decided to offer to the Metropolitan Asylums Board, on terms to be arranged hereafter, their workhouses at Edmonton and Sheffield Street. The former is a large building with certified accommodation for 1,098 inmates, the latter is much smaller, and has beds for sixty-eight inmates only. The Guardians decided at the same time to offer to sell their Poland Street Workhouse, which has accommodation for 573 inmates, to the Westminster City Council for the erection thereon of workmen's dwellings. The staff and establishments committee of the Guardians reported that all the inmates of these institutions could be accommodated in the Guardians' other workhouses except 200 or so, which could be boarded out in Chelsea and Kensington. It was estimated that a saving of £30,000 to £45,000 annually would be effected by these arrangements. As regards Greenwich the offer of the Metropolitan Asylums Board has been referred to a committee.

The advantages of these proposals are obvious. Not only will there be a great saving to the two individual Unions concerned, but the ratepayers of the whole of London will benefit from the fact that the Metropolitan Asylums Board will be able to acquire the additional accommodation required at a small cost, as compared with what would be incurred if new buildings were to be provided. The great disadvantage is that the scheme deals with the institutional relief of the poor of London in a piecemeal manner. The Metropolitan Asylums Board and the two Unions concerned naturally consider only their own special requirements. But the problem is a much bigger one. The arbitrary division of London into its present twenty-eight Unions can no longer be considered an economic or politic one, at any rate so far as concerns the provision of indoor relief, for its sick and able-bodied poor. The necessity for classification by institutions and for specialised treatment of their inmates is becoming more urgent every day. A statesmanlike solution of the problems offered can only be effected by one central body able to deal with London as a whole. An object-lesson of what is possible by centralisation has been afforded in the last two years by the results obtained by taking the administration of the casual wards of London from the Guardians and handing it over to the Metropolitan Asylums Board. It has been found possible to close a number of the casual wards and to reduce very considerably the number of vagrant poor in the Metropolis. Similar and even more important results would undoubtedly follow from an extension of the same principle to infirmaries and workhouses.

